# Clinical features of COVID-19 infection in patients with myasthenia gravis: a real-world retrospective study

**DOI:** 10.3389/fpubh.2024.1421211

**Published:** 2024-08-27

**Authors:** Hui-Ning Li, Xiao-Na Xu, Ying-Hui Qin, Rui Liu, Wen-Yue Guo, Xiao-Yu Huang, Mo-Li Fan, Lin-Jie Zhang, Yuan Qi, Chao Zhang, Li Yang, Fu-Dong Shi, Chun-Sheng Yang

**Affiliations:** ^1^Department of Neurology, Tianjin Neurological Institute, Tianjin Medical University General Hospital, Tianjin, China; ^2^China National Clinical Research Center for Neurological Diseases, Beijing Tiantan Hospital, Capital Medical University, Beijing, China

**Keywords:** myasthenia gravis, coronavirus-19, immunosuppression, neuromuscular junction disorders, viral immunology

## Abstract

**Objective:**

We investigated the risk factors associated with severe or critical Coronavirus disease 2019 (COVID-19) infection due to the Omicron variant in patients with myasthenia gravis (MG) and determined the potential effect of COVID-19 on myasthenic exacerbation during the Omicron pandemic.

**Methods:**

This retrospective study included 287 patients with MG in Tianjin, China. Clinical data of the patients were collected using electronic questionnaires, databases, and clinical records.

**Results:**

The overall infection rate was 84.7%. Advanced age, comorbidities, generalized phenotype, and MG instability were drivers of COVID-19 severity, and post-COVID-19 myasthenic exacerbation. The concurrent use of a steroid-sparing agent did not affect COVID-19 susceptibility or severity. It did lower the risk of myasthenic exacerbation after COVID-19 infection. Patients with severe COVID-19 experienced myasthenic exacerbation earlier than patients with non-severe infection (*p* < 0.001). The severity of COVID-19 (Hazards Ratio = 3.04, 95% CI: 1.41–6.54, *p* = 0.004) and the clinical phenotype (Hazards Ratio = 3.29, 95% CI: 1.63–6.63, *p* < 0.001) emerged as independent risk factors for early MG exacerbation.

**Conclusion:**

Generally, patients with MG appear to be susceptible to the Omicron strains. Immunotherapy for MG did not increase COVID-19 susceptibility or severity. We do not advocate an immediate cessation of ongoing immunosuppressive treatments once a COVID-19 infection is diagnosed. Instead, a judicious evaluation of the risks and benefits, tailored to each individual, is recommended.

## 1 Introduction

Coronavirus disease 2019 (COVID-19) is caused by a novel coronavirus known as severe acute respiratory syndrome coronavirus 2 (SARS-CoV-2). Symptoms are diverse but typically include fever, cough, respiratory symptoms, diarrhea, dysgeusia, and anosmia (1). The severity of the disease ranges from mild to severe, and the virus has the potential to cause pneumonia, acute respiratory distress syndrome, and death. Neurological complications of COVID-19 include headache, myalgia, encephalopathy, and cerebrovascular disease (2–4). Immune-mediated neuropathy (5, 6) and neuromuscular disorders (7) have also been observed, likely resulting from the disruption of immunological self-tolerance triggered by SARS-CoV-2 infection.

Patients with myasthenia gravis (MG) may be particularly vulnerable in the era of the COVID-19 pandemic, partially because of respiratory muscle weakness (8). In addition, pyridostigmine may increase mucus secretion and render airway management intractable. Patients who are moderately or severely immunocompromised have a greater risk of a prolonged COVID-19 clinical course (1). There is a dynamic feedback loop in which myasthenic symptoms and infection exacerbate each other (9). Bystander activation of the pre-existing inflammatory environment during infections might accelerate autoimmune responses (10). Exposure to certain drugs, such as azithromycin (11) or hydroxychloroquine (12, 13), also worsens myasthenia gravis. These medications could contribute to the exacerbation caused by the infection. Despite numerous studies on the association between COVID-19 and MG (14–17), the clinical courses and outcomes of patients with MG and COVID-19 are highly variable. Further large-scale studies are needed to delineate the best practices and outcome determinants in this unique population.

An international group of neuromuscular physicians developed initial guidelines for managing MG during the pandemic based on their collective experience with viral illnesses in this specific patient population (18). Nonetheless, evidence for continuing immunosuppressive medications is lacking, and the available data from the international COVID-19 Associated Risks and Effects in Myasthenia Gravis (CARE-MG) registry are limited (19). Thus, the risk factors associated with COVID-19 susceptibility and severity need to be identified in MG patients. However, the effect of COVID-19 on MG stability remains to be elucidated. Therefore, real-world evidence is necessary to specify the management recommendations for patients with both MG and COVID-19.

## 2 Materials and methods

### 2.1 Population of interest

**Figure 1:**
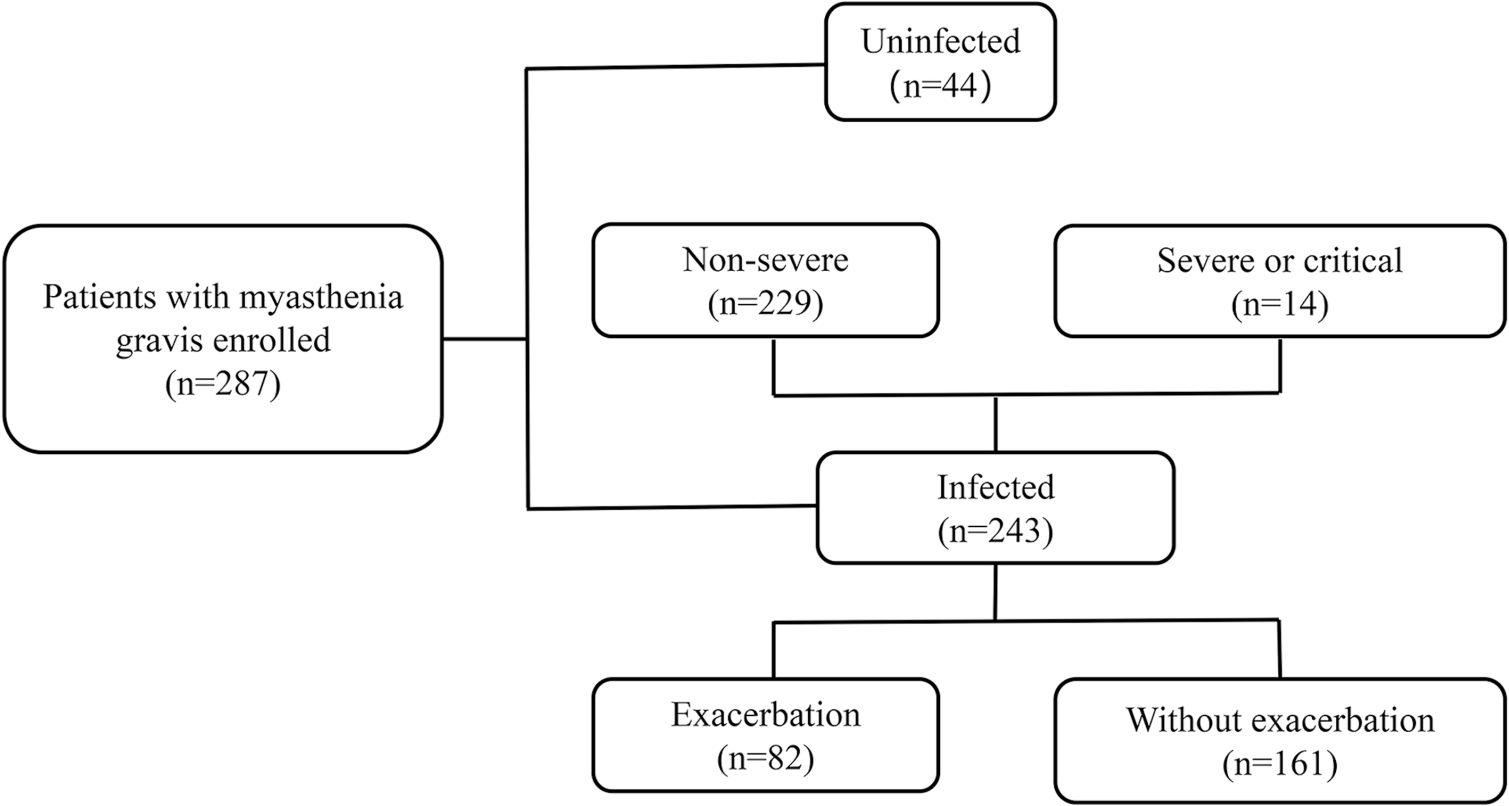
Cohort details according to dual outcome endpoints. The patients were retrospectively enrolled between December 2022 and March 2023.

This was a single-center retrospective study of patients diagnosed with MG in the neurology department of Tianjin Medical University General Hospital (Figure 1). The diagnosis of MG was based on fluctuating skeletal muscle weakness and at least one of the following three conditions (20): (1) positive neostigmine testing; (2) seropositivity for antibodies against acetylcholine receptors (AChRs), muscle-specific kinase (MuSK), or lipoprotein-related protein 4; (3) abnormal repetitive nerve stimulation (RNS) (3 Hz RNS was applied to the facial, ulnar, axillary, and accessory nerves, the amplitude of the compound muscle action potential decreased by >10%).

According to the Diagnosis and Treatment Plan for Novel Coronavirus Infection (Trial Version 10) outlined by the National Health Commission of China, the diagnosis of COVID-19 was confirmed by a positive PCR test or an antigen test for SARS-CoV-2 conducted on a nasopharyngeal swab. COVID-19 was considered probable if the patient met at least one of the following criteria: (1) a history of epidemiological contact; (2) presentation of critical signs such as fever, cough, respiratory symptoms, diarrhea, and reduction of smell and taste sensation; and (3) suggestive abnormalities were observed on a thoracic computed tomography (CT) scan. Severe COVID-19 was defined as the presence of at least one of the following: (1) respiratory rate ≥ 30 breaths per minute; (2) oxygen saturation ≤ 93% in room air; (3) significant progression in >50% of the pneumonia lesions on thoracic CT scans within 24–48 h. Critical COVID-19 was defined as respiratory distress syndrome (ARDS) requiring mechanical ventilation (invasive or noninvasive) or other organ failure requiring admission to the intensive care unit. Non-severe COVID-19 was defined as the absence of criteria indicative of severe or critical COVID-19.

### 2.2 Data collection

All COVID-19-related data were measured between December 22, 2022 and March 23, 2023. China significantly eased COVID-19 measures on December 7, 2022, after which China had an Omicron wave, as expected. The new infection rate of COVID-19 peaked from late 2022 to early 2023, mainly attributed to the Omicron variant.^1^ Predefined electronic questionnaires were sent to participants registered in our MG database in Tianjin Medical University General Hospital. The questionnaires collected information on each participant’s infection and exacerbation. The clinical characteristics of the participants were retrieved from the database. Treatments for COVID-19 and MG were recorded from inpatient and outpatient clinical records. Patients reported myasthenic exacerbation during COVID-19 using the Patient Global Impression of Change (PGI-C) scale, which is a seven-point Likert scale of patient-reported outcomes (PROs). These potential exacerbations were confirmed by observing a minimum two-point increase in the activities of daily living (ADL) scales (21, 22). The institutional Ethics Committee approved this study (approval number IRB2023-YX-215-01). Clinical data were analyzed anonymously. Informed consent was waived due to the retrospective nature of this observational study.

Medications for MG included steroids and non-steroid immunotherapy. Steroid regimens were classified as low-dose regimens (≤20 mg) and high-dose regimens (>20 mg). Nonspecific nonsteroidal immunosuppressants (NSIS) included tacrolimus, azathioprine, mycophenolate mofetil, and cyclophosphamide. Biological monoclonal antibodies (mAbs) included interleukin (IL)-6 inhibitors, B-cell–depleting therapy (BCDT), and neonatal Fc receptor (FcRn) inhibitors.

### 2.3 Outcome measurements

The dual-outcome endpoints were COVID-19 infection and myasthenic exacerbations after COVID-19 infection. COVID-19 was diagnosed according to the “Diagnosis and Treatment Plan for Novel Coronavirus Infection (Trial Version 10)” outlined by the National Health Commission of China. Disease exacerbation was defined as additional symptoms or exacerbation of existing symptoms in at least one of the five muscle groups (ocular, bulbar, limb, cervical, and respiratory muscles) within 30 days of infection with an increase in the ADL score of ≥2 points. A change in ADL score of 2 points was adopted based on the established minimal clinically important difference (MCID), the smallest outcome change that has clinical significance (23). Sensitivity/specificity analysis revealed that a two-point change of ADL has the best trade-off attributes to predict clinical improvement or exacerbation (21). ADL MCID values provide clinically relevant information and have been applied in multiple MG clinical trials (such as NCT03971422 and NCT03669588).

### 2.4 Statistical analysis

The normality of the data distributions was estimated using the Shapiro–Wilk method. Quantitative data meeting the normal distribution are presented as the mean ± standard deviation (SD), and the corresponding data were compared using the Student’s *t*-test. Quantitative data without a normal distribution are expressed as the median and interquartile range (IQR). The corresponding data were compared using the rank-sum test. Fisher’s exact test and the chi-squared test were used to compare categorical variables, which are presented as counts and percentages. Logistic regression analysis was used to identify potential factors contributing to COVID-19 susceptibility and severity. Firth’s penalized likelihood logistic regression was adopted to solve the separation problem (24). Kaplan–Meier curves were used to visualize the timeline over the dual outcome endpoints. Cox proportional hazards models were used to identify factors influencing myasthenic exacerbation post–COVID-19 infection. Independent variables with *p* values <0.2 in the univariate model were included in the multivariate ordinal model, where a *p* value <0.05 was considered statistically significant. The goodness-of-fit of the models was also verified. Statistical analysis and data processing were performed in R version 4.3.1 (R Foundation), using the “logistf” (version 1.26.0) and “survival” packages (version 3.5-5).

## 3 Results

### 3.1 Omicron susceptibility

Although widespread vaccination efforts were implemented, the infection rate was high during the wave of SARS-CoV-2 infection caused by the Omicron variant. In our cohort, 243/287 (84.7%) individuals were infected, while 44/287 (15.3%) remained uninfected (Figure 1). The demographic and clinical characteristics of the MG patients were comparable between the two groups, including age at diagnosis, disease duration, serotype, and thymus status (Table 1). We investigated the susceptibility to COVID-19 in individuals with several related or common comorbidities, including pulmonary, cardiovascular, metabolic, oncologic, or autoimmune complications. None of these comorbidities, including asthma, chronic obstructive pulmonary disease, or pulmonary fibrosis, increased the susceptibility to COVID-19. In terms of clinical phenotype, we dichotomized our participants into ocular MG (OMG) and generalized MG (GMG), as the underlying pathogenesis and treatment principles between the two are different. In our cohort, 158 (65.0%) patients were GMG in the infected group and 24 (54.5%) in the uninfected group. There was no significant difference in the clinical phenotype of MG between the infected and uninfected group (*p* = 0.184; Table 1).

**Table 1:** Comparison between infected and uninfected patients.

	Infected	Uninfected	*p*-value
	(*n* = 243)	(*n* = 44)	
Age, year, mean (SD)	58.9 (±0.9)	60.0 (±2.1)	0.643
Sex			0.860
Male, No. (%)	125 (51.4)	22 (50.0)	
Female, No. (%)	118 (48.6)	22 (50.0)	
MG duration, year, median (P_25_, P_75_)	2 (1, 3)	2 (1, 2.75)	0.895
MG subgroups			0.081
Early-onset, No. (%)	83 (34.2)	9 (20.5)	
Late-onset, No. (%)	160 (65.8)	35 (79.5)	
Clinical phenotype			0.184
OMG, No. (%)	85 (35.0)	20 (45.5)	
GMG, No. (%)	158 (65.0)	24 (54.5)	
Comorbidities			0.446
Yes, No. (%)	113 (46.5)	27 (61.4)	
No, No. (%)	130 (53.5)	17 (38.6)	
Antibodies			0.135
AChR-Ab (+), No. (%)	196 (80.7)	35 (79.5)	
MuSK-Ab (+), No. (%)	11 (4.5)	5 (11.4)	
Seronegative, No. (%)	36 (14.8)	4 (9.1)	
Thymus			0.663
Normal, No. (%)	186 (76.5)	35 (79.5)	
Abnormality^a^, No. (%)	57 (23.5)	9 (20.5)	
Prednisone alone, mg			
0^#^, No. (%)	96 (60.0)	16 (53.3)	
≤20, No. (%)	46 (28.8)	11 (36.7)	0.488
>20, No. (%)	18 (11.2)	3 (10.0)	1.000
Additional immunotherapy			
Absence^#^, No. (%)	160 (65.3)	30 (68.2)	
NSIS^b^, No. (%)	56 (22.9)	10 (22.7)	1.000
mAbs^c^, No. (%)	29 (11.8)	4 (9.1)	0.794

^#^The reference group and two other subgroups were compared. SD, Standard deviation; COVID-19, Coronavirus disease 2019; MG, Myasthenia gravis; OMG, Ocular myasthenia gravis; GMG, Generalized myasthenia gravis; AChR, Acetylcholine receptor; MuSK, Muscle-specific tyrosine kinase.^a^Thymic abnormalities refer to thymic hyperplasia or thymoma and whether surgical resection is included.^b^NSIS, Nonsteroidal immunosuppressants, including tacrolimus, azathioprine, mycophenolate mofetil, and cyclophosphamide.^c^mAbs, biological monoclonal antibodies, B cell deletion agents, IL-6 blockers, and FcRn inhibitors.

Of all the patients, 78 were administered corticosteroids alone. No significant differences in infection rates were observed in either the low dose group (≤20 mg) or the high dose group (>20 mg) compared to the corticosteroid-free group (*p* = 0.488 and *p* = 1.000, respectively; Table 1). In addition, we tested the association between COVID-19 and various immunosuppressants and immunomodulators. Sixty-six (23.0%) patients were on NSIS, including tacrolimus, azathioprine, mycophenolate, mofetil, and cyclophosphamide, and 33 (11.5%) patients were treated with biologic therapies (23 with BCDT, eight with IL-6 inhibitors, and two with FcRn inhibitors). Patients on these regimens did not bear the additional burden of Omicron susceptibility (*p* = 1.000 for NSIS and *p* = 0.794 for mAb therapy, respectively; Table 1).

### 3.2 COVID-19 severity

**Figure 2:**
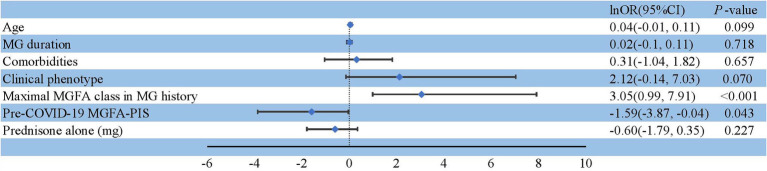
Risk factors for a severe or critical course of COVID-19.

Among the 243 patients with MG infected during the Omicron wave, 229 were diagnosed with non-severe infection, 14 with severe or critical infection (Table 2), and five required ventilation (Supplementary Table 1). Thirty-six patients were hospitalized, and three individuals died primarily due to COVID-related lung injuries and partially as a result of MG exacerbation (Table 3). The severity of COVID-19 was affected by age, comorbidities, clinical phenotype, maximal Myasthenia Gravis Foundation of America (MGFA) classification in MG history, and pre-COVID-19 MGFA post-intervention status (MGFA-PIS). Patients who were older or had chronic comorbidities had a greater incidence of severe COVID-19 infection (*p* = 0.009 and *p* = 0.013, respectively). In addition, patients with GMG tended to experience a severe course of COVID-19 (*p* = 0.005). Moreover, patients with a high MGFA classification previously or a worse pre-COVID-19 MGFA-PIS were at a greater risk of severe or critical infection (*p* < 0.001 and *p* = 0.013, respectively; Table 2). We assessed whether immunotherapy, encompassing different intensities of oral corticosteroids, nonspecific NSIS, or mAbs, could precipitate a severe to critical infection course. Consistent with the findings regarding Omicron susceptibility (Table 1), COVID-19 severity was not adversely affected in our immunosuppressed population (Table 2). Only one patient with BCDT developed severe pneumonia (Supplementary Table 1). After treatment with intravenous methylprednisolone (80 mg) and azvudine, a reverse transcriptase inhibitor, the patient recovered and was discharged 14 days after admission. A previous high MGFA classification (OR = 21.20, 95% CI: 2.69–2734.28, *p* < 0.001) and worse pre-COVID-19 MGFA-PIS (OR = 0.20, 95% CI: 0.02–0.96, *p* = 0.043) were independent risk factors for severe or critical COVID-19 infection (Figure 2).

**Table 2:** Comparison between patients with non-severe and severe or critical COVID-19.

	Non-severe	Severe or critical	*p*-value
	(*n* = 229)	(*n* = 14)	
Age, year, mean (SD)	58.3 (±0.9)	68.6 (±3.5)	0.009
Sex			0.660
Male, No. (%)	117 (51.1)	8 (57.1)	
Female, No. (%)	112 (48.9)	6 (42.9)	
MG duration, year, median (P_25_, P_75_)	2 (1, 5)	4.5 (2, 8.5)	0.118
MG subgroups			0.185
Early-onset, No. (%)	81 (35.4)	2 (14.3)	
Late-onset, No. (%)	148 (64.6)	12 (85.7)	
Clinical phenotype			0.005
OMG, No. (%)	85 (37.1)	0 (0.0)	
GMG, No. (%)	144 (62.9)	14 (100.0)	
Comorbidities			0.013
Yes, No. (%)	102 (44.5)	11 (78.6)	
No, No. (%)	127 (55.5)	3 (21.4)	
Antibodies			1.000
AChR-Ab (+), No. (%)	184 (80.3)	12 (85.7)	
MuSK-Ab (+), No. (%)	11 (4.8)	0 (0.0)	
Seronegative, No. (%)	34 (14.8)	2 (14.3)	
Thymus			0.246
Normal, No. (%)	173 (75.5)	13 (92.9)	
Abnormality^a^, No. (%)	56 (24.5)	1 (7.1)	
Maximal MGFA class in MG history			<0.001
I–II, No. (%)	138 (60.3)	0 (0.0)	
III–V, No. (%)	91 (39.7)	14 (100.0)	
Pre-COVID-19 MGFA-PIS			0.013
MMS or better ^b^, No. (%)	68 (29.7)	0 (0.0)	
Inferior to MMS, No. (%)	161 (70.3)	14 (100.0)	
Prednisone alone, mg			
0^#^, No. (%)	91 (61.1)	5 (45.5)	
≤20, No. (%)	40 (26.8)	6 (54.5)	0.176
>20, No. (%)	18 (12.1)	0 (0.0)	1.000
Additional immunotherapy			
Absence^#^, No. (%)	149 (64.5)	11 (78.6)	
NSIS^c^, No. (%)	54 (23.4)	2 (14.3)	0.522
mAbs^d^, No. (%)	28 (12.1)	1 (7.1)	1.000

^#^The reference group and two other subgroups were compared.^a^Thymic abnormalities refer to thymic hyperplasia or thymoma and whether surgical resection is included.^b^MMS or better, including complete response (CSR) or drug response (PR).^c^NSIS, Nonsteroidal immunosuppressants, including tacrolimus, azathioprine, mycophenolate mofetil, and cyclophosphamide.^d^mAbs, biological monoclonal antibodies, B cell deletion agents, IL-6 blockers, and FcRn inhibitors.

SD, Standard deviation. MGFA, Myasthenia Gravis Foundation of America; PIS, Post-intervention status; COVID-19, Coronavirus disease 2019; MG, Myasthenia gravis; OMG, Ocular myasthenia gravis; GMG, Generalized myasthenia gravis; AChR, Acetylcholine receptor; MuSK, Muscle-specific tyrosine kinase.

**Table 3:** Clinical characteristics about myasthenic exacerbation.

Characteristics	
**Case of exacerbation, No. (%)**	
Non-severe	68/82 (82.9)
Severe or critical	14/82 (17.1)
**Duration to exacerbation, d, median (P_25_, P_75_)**	
Non-serve	20 (10, 30)
Severe or critical	7 (3.8, 10)
Age, years, mean (SD)	61.4 (±13.4)
MG duration, years, median (P_25_, P_75_)	2 (1, 5.3)
**Antibodies, No. (%)**	
AChR-Ab (+)	67/82(81.7)
MuSK-Ab (+)	0/82(0)
Seronegative	15/82 (18.3)
**Hospitalized, No. (%)**	
Yes	36/82 (43.9)
No	46/82 (56.1)
Hospitalization duration, day, median (P_25_, P_75_)	11 (9, 14)
ICU admission, No. (%)	5/82 (6.1)
**Mechanical ventilation, No. (%)**	
Non-invasive mechanical ventilation	2/82 (2.4)
Invasive mechanical ventilation	4/82 (4.9)
**MGFA class at exacerbation, No. (%)**	
I	26/82 (31.7)
II	31/82 (37.8)
III	18/82 (21.9)
IV	2/82 (2.4)
V	5/82 (6.0)
Gastric tube, No. (%)	6/82 (7.3)
**The use of antibiotics, No. (%)**	**25/82 (30.5)**
Azithromycin	1/81 (1.2)
Cefuroxime	1/82 (1.2)
Piperacillin-tazobactam	11/82 (3.7)
Cefoxitin	11/82 (13.4)
Unnamed	1/81 (1.2)
**Affected muscles, No. (%)**	
Ocular muscles	45/82 (54.9)
Bulbar muscles	20/82 (24.4)
Limb muscles	20/82 (24.4)
Cervical muscles	5/82 (6.1)
Respiratory muscles	16/82 (19.5)
**Treatment, No. (%)**	
Untreated	9/82 (10.9)
IVIG	27/82 (32.9)
IVMP	32/82 (39.0)
Oral medications adjusted	35/82 (42.7)
Death, No. (%)	3/82 (3.7)

SD, Standard deviation; MG, Myasthenia gravis; AChR, Acetylcholine receptor; MuSK, Muscle-specific tyrosine kinase; COVID-19, Coronavirus disease 2019; MGFA, Myasthenia Gravis Foundation of America; ICU, Intensive care unit; IVIG, Intravenous immunoglobulin; IVMP, Intravenous methylprednisolone.

### 3.3 Myasthenic exacerbations

**Figure 3:**
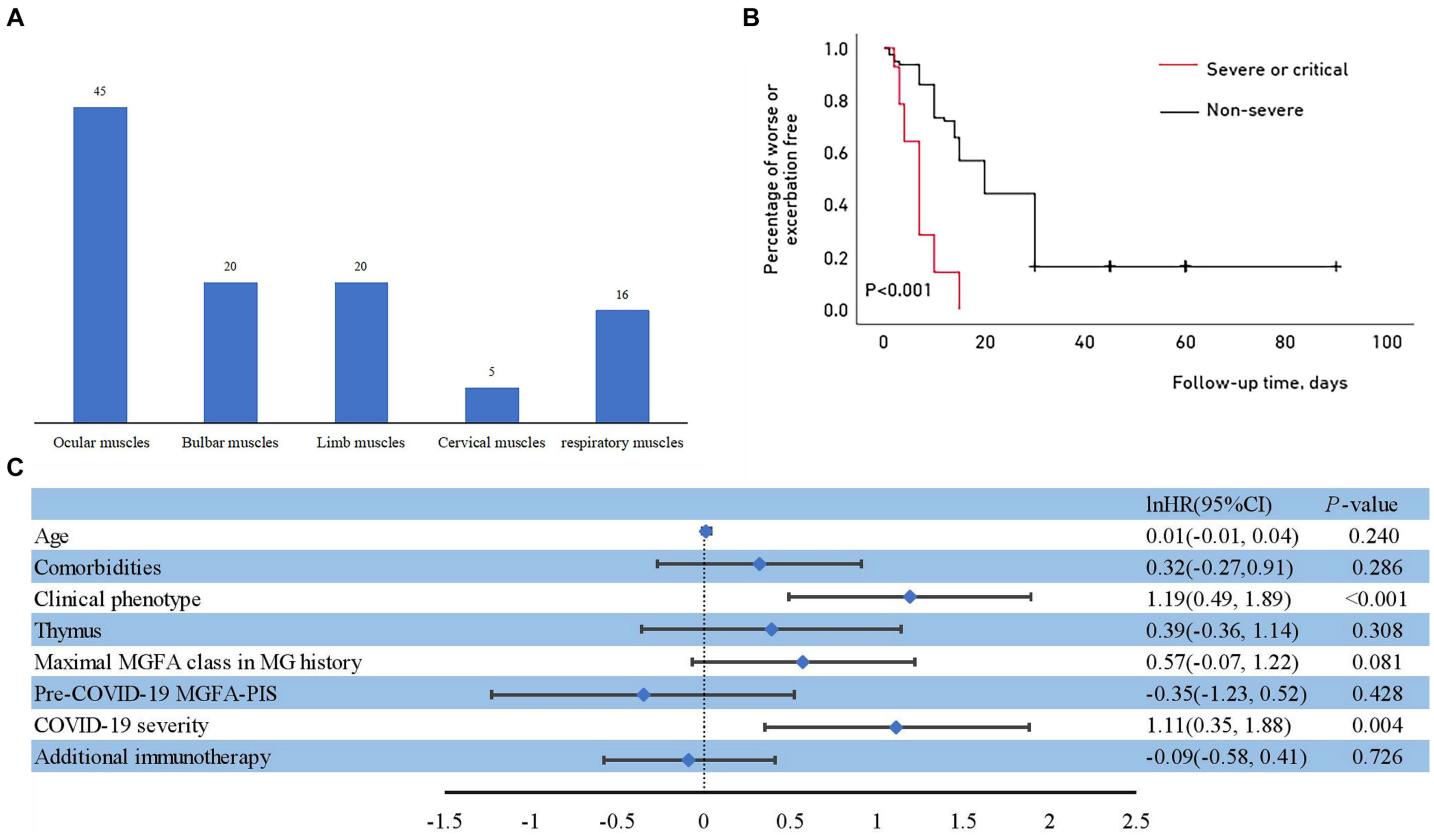
**(A)** Muscle groups involved during myasthenic exacerbation. **(B)** Outcomes of myasthenic exacerbation with different severities of COVID-19. Kaplan–Meier curve for the cumulative incidence of myasthenic exacerbation for the non-severe group compared with the severe or critical group. **(C)** Risk factors for myasthenic exacerbation after COVID-19.

Eighty-two patients (82/243, 33.7%) experienced myasthenic exacerbations during the course of COVID-19 (Figure 1), including all 14 patients with severe or critical COVID-19 and 68 (29.7%) patients with a non-severe COVID-19 course. Of these patients, 36 (43.9%) were admitted to the hospital, and five were admitted to the intensive care unit. The majority of exacerbations were related to the ocular muscles, with respiratory muscles affected in 16 patients (Figure 3A). Of the patients with worsened MG, 27 (32.9%) received intravenous immunoglobulin (IVIG) as rescue therapy. Two patients required noninvasive mechanical ventilation, and four required invasive ventilation. One patient was treated with both noninvasive and invasive mechanical ventilation. Although antibiotics for bacterial superinfections in patients with MG are used with caution, one patient experienced exacerbation after receiving azithromycin. One patient who withdrew from prednisone treatment during COVID-19 infection experienced global weakness in various muscle groups, including the cervical, bulbar, and extremities (Table 3). The myasthenic exacerbations were affected by age (*p* = 0.046, Table 4), clinical phenotype (*p* = 0.029, Table 4), comorbidities (*p* = 0.007, Table 4), maximal MGFA classification in MG history (*p* = 0.027, Table 4), and pre-COVID-19 MGFA-PIS (*p* < 0.001).

**Table 4:** Comparison between patients with and without myasthenia gravis exacerbation.

	Exacerbation	Without exacerbation	*p*-value
	(*n* = 82)	(*n* = 161)	
Age, year, mean (SD)	61.4 (±1.5)	57.6 (±1.2)	0.046
Sex			0.204
Male, No. (%)	37 (45.1)	88 (54.7)	
Female, No. (%)	45 (54.9)	73 (45.3)	
MG duration, year, median (P_25_, P_75_)	2 (1, 6.3)	3 (1, 5)	0.945
MG subgroups, No. (%)			0.115
Early-onset, No. (%)	22 (26.8)	61 (37.9)	
Late-onset, No. (%)	60 (73.2)	100 (61.1)	
Clinical phenotype			0.029
OMG, No. (%)	21 (25.6)	64 (39.8)	
GMG, No. (%)	61 (74.4)	97 (60.2)	
Comorbidities			0.007
Yes, No. (%)	48 (58.5)	65 (40.4)	
No, No. (%)	34 (41.5)	96 (59.6)	
Antibodies			0.657
AChR-Ab (+), No. (%)	66 (80.5)	130 (80.8)	
MuSK-Ab (+), No. (%)	5 (6.1)	6 (3.7)	
Seronegative, No. (%)	11 (13.4)	25 (15.5)	
Thymus			0.092
Normal, No. (%)	68 (82.9)	118 (73.3)	
Abnormality^a^, No. (%)	14 (17.1)	43(26.7)	
Maximal MGFA class in MG history			0.027
I–II, No. (%)	38 (46.3)	100 (62.1)	
III–V, No. (%)	44 (53.7)	61 (37.9)	
Pre–COVID-19 MGFA-PIS			<0.001
MMS or better^b^, No. (%)	8 (9.8)	60 (37.3)	
Inferior to MMS, No. (%)	74 (90.2)	101 (62.7)	
Prednisone alone, mg			
0^#^, No. (%)	40 (60.6)	56 (59.6)	
≤20, No. (%)	21 (31.8)	25 (26.6)	0.789
>20, No. (%)	5 (7.6)	13 (13.8)	0.306
Additional immunotherapy			
Absence^#^, No. (%)	66 (79.5)	94 (58.0)	
NSIS^c^, No. (%)	12 (14.5)	44 (27.2)	0.013
mAbs^d^, No. (%)	5 (6.0)	24 (14.8)	0.009
COVID-19 severity			<0.001
Non-severe, No. (%)	68 (82.9)	161 (100.0)	
Severe or critical, No. (%)	14 (17.1)	0 (0.0)	

^#^The reference group and two other subgroups were compared.

SD, Standard deviation; MGFA, Myasthenia Gravis Foundation of America; PIS, Post-intervention status; COVID-19, Coronavirus disease 2019; MG, Myasthenia gravis; OMG, Ocular myasthenia gravis; GMG, Generalized myasthenia gravis; AChR, Acetylcholine receptor; MuSK, Muscle-specific tyrosine kinase.

^a^Thymic abnormalities refer to thymic hyperplasia or thymoma and whether surgical resection is included.^b^MMS or better, including complete response (CSR) or drug response (PR).^c^NSIS, nonsteroidal immunosuppressants, including tacrolimus, azathioprine, mycophenolate mofetil, and cyclophosphamide.^d^mAbs, biological monoclonal antibodies, including B cell deletion agents, IL-6 blockers, and FcRn inhibitors.

In addition to not affecting COVID-19 susceptibility and severity in our cohort, the concomitant administration of a steroid-sparing agent lowered the risk of MG exacerbation after COVID-19 (*p* = 0.013 for NSIS and *p* = 0.009 for mAb; Table 4). No differences were detected in patients receiving higher or lower doses of steroids alone compared to the reference group (*p* = 0.789 and *p* = 0.306, respectively; Table 4). The Kaplan–Meier analysis showed that although the overall proportion of patients who remained stable after COVID-19 decreased over the observation period, patients with severe to critical infection experienced exacerbation earlier than those with non-severe infection (Figure 3B). The median time to exacerbation was 7 days, which was significantly shorter than that in the non-severe infection group (Table 3). The multivariate Cox proportional hazard regression model identified that COVID-19 severity and clinical phenotype were independent risk factors for early MG exacerbation (Hazards Ratio = 3.04, 95% CI: 1.41–6.54, *p* = 0.004; Hazards Ratio = 3.29, 95% CI: 1.63–6.63, *p* < 0.001, respectively; Figure 3C).

## 4 Discussion

To our knowledge, our study is the largest cohort conducted on patients with both MG and COVID-19 to date in Northern China. Patients with advanced age, comorbidities, generalized phenotype, a previous high MGFA classification or worse pre-COVID-19 MGFA-PIS are more prone to severe COVID-19, which independently emerged as a risk factor for post-COVID-19 myasthenic exacerbation. Some of these common risk factors were also identified in other reports (16, 25). A case–control study of 311 patients with MG who developed COVID-19 showed that the baseline MG-ADL, duration of symptomatic COVID-19, and GMG are risk factors for exacerbation in MG patients with COVID-19 (26). Advanced age and comorbidities are well-recognized as aggravating factors for COVID-19 infection (27). Previous high MGFA classification and worse pre-COVID-19 MGFA-PIS implied unsatisfactory MG symptom control. The stronger immune perturbations underlying the instability of MG could lead to deterioration of respiratory parameters and pneumonia progression later in the course of COVID-19. Patients with inadequate control of MG symptom may need closer monitoring during the course of COVID-19 infection. These results are important for establishing evidence-based guidelines for managing patients with MG during the COVID-19 pandemic.

Chronic immunosuppression is reported as a risk factor for a severe course of COVID-19 (28). However, we did not observe an increase in COVID-19 susceptibility or severity in our cohort. Different immunosuppression intensities and durations in real-world settings may have contributed to this discrepancy. Cytokine storms are a major problem in COVID-19 (29). Corticosteroid therapy has been recommended for severe COVID-19 when the criteria for ARDS are fulfilled (1). In addition, tocilizumab has been investigated as a possible anti-inflammatory medication for cytokine storms caused by COVID-19 (30–32). Immunosuppressants can moderate the cytokine storm, thereby playing a positive role in COVID-19 management (33). Patients with GMG may face a heightened risk of relapse if they discontinue immunotherapeutic treatments. Some drug washouts may require an extended duration, and restoring the response to these drugs takes several months. The International MG/COVID-19 Working Group also recommends that the adjustment of management should be individualized (18). Based on our own data, we do not recommend stopping current immunotherapeutic agents abruptly once an infection is established.

Patients undergoing recent BCDT have a poor ability to develop anti–SARS-CoV-2 antibodies (34), possibly leading to a severe and prolonged COVID-19 course (35). The European Society of Neurology (36) and International MG/COVID-19 Working Group (18) agreed that it might be better to suspend the use of B-cell–depleting agents such as rituximab. Rituximab, a chimeric monoclonal antibody, targets CD20-positive B-lymphocytes, which play critical roles in both COVID-19 and MG. Numerous studies on multiple sclerosis showed that anti-CD20 therapies were significantly associated with COVID-19 severity (37, 38). However, other studies did not report additional unfavorable outcomes in patients undergoing BCDT (39, 40). Patients receiving rituximab in our study did not exhibit a greater risk of contracting COVID-19 or experiencing a severe course, partially because of lower doses and extended dosing intervals in the rituximab regimen (41) we adopted. We assume that immunological factors other than B-cell–mediated antibody responses are preserved for the control of COVID-19. A comprehensive immune profiling of patients with MG receiving rituximab corroborated our speculation (42). Despite the impaired B cell and humoral response, patients under rituximab showed an intact innate, CD8 T-cell and IFN-γ specific CD4+ and CD8+ T-cell response after infection and vaccination.

Due to its immunomodulatory effect, IVIG therapy can be beneficial for patients with COVID-19 and acute myasthenic exacerbation (43). However, the use of IVIG should be tailored to individual needs, and indiscriminate use should be avoided. Evidence suggests that IVIG might increase the risk of thrombosis, including multifocal stroke in COVID-19 (44). We exercised extreme caution regarding the hypercoagulable state during COVID-19. Anticoagulant therapies were given to high-risk patients (Supplementary Table 1). We did not observe any thromboembolic complications associated with IVIG treatment.

Eighty-two patients (33.7%) experienced myasthenic exacerbations in our cohort during the course of COVID-19, which is consistent with that observed after COVID-19 in the CARE-MG registry (19). Myasthenic exacerbation, especially the weakening of respiratory muscles, should be distinguished when chest imaging findings do not align with the severity of respiratory insufficiency. The triggers of myasthenic exacerbations are multifactorial, although infections are considered as the most common cause. In multivariate analysis, only COVID-19 severity and clinical phenotype were independent risk factors for myasthenic exacerbation. We speculated that the impact of COVID-19 severity is so high that it probably nullifies the effect of other risk factors of myasthenic exacerbation such as age, comorbidities, previous high MGFA classification, and worse pre-COVID-19 MGFA-PIS. The autoimmune or parainfectious response elicited by SARS-CoV-2 infection appears to be active up to 30 days after infection. Thus, we stopped measuring MG exacerbations at 30 days post infection. This duration is also consistent with the latency of myasthenic symptoms to occur or exacerbate after vaccinations as observed in other reports (45). We consider the myasthenic exacerbation that occurred within this timeframe was more likely to have been induced by SARS-CoV-2 infection than other incentive. Myasthenic exacerbation occurred with a median time of 7 days after SARS-CoV-2 infection in severe or critical patients in our cohort, consistent with the time from infection to symptoms that has been observed in other neurological disorders (6). Patients with severe COVID-19 in our cohort experienced myasthenic exacerbation earlier than patients with non-severe infection. A latency <1 week typically could represent the effect of the pre-existent memory B cells that produce low-affinity antibodies involved in the non-specific immune response; on the contrary, a latency >1 week could be the influence of adaptive immunity (46).

Besides myasthenic exacerbations, COVID-19-induced MG is more noteworthy. There were also three cases of new-onset MG after SARS-CoV-2 infection in our cohort. Due to the rarity of this situation, multicenter longitudinal studies are needed to recruit enough participants. In addition, it remains controversial if MG developed as a new-onset disease or was pre-existent subclinically. Mechanically, some authors speculated that new-onset MG after COVID-19 could be explained by molecular mimicry (7, 47) or by the breakdown of self-tolerance mechanisms as a consequence of the infection (48), as implicated in other neurological autoimmune disorders after infection. However, there appears to be no apparent structural match between subunits of acetylcholine receptors and SARS-CoV-2 proteins. Others supported the possibility that COVID-19 could have triggered latent MG (49). Nevertheless, evidence from fundamental studies is warranted to analyze the immunological characteristics of MG in the setting of COVID-19.

Our study had a few limitations. First, this was a single-center retrospective study. Selection bias was inevitable due to the inherent limitations of retrospective studies. MG itself is highly heterogeneous, with various serological and clinical features. The management of MG was performed at the discretion of the treating physicians. Second, despite the overall large size of the cohort, the relatively small number of patients with a severe to critical course of COVID-19 could have influenced the outcomes of this study compared to those of other groups. Finally, this study did not discuss the effects of different doses and durations of concomitant steroid-sparing agents on COVID-19 and myasthenic exacerbation; thus, more cases and refined stratification are needed for further discussion.

## Data Availability

The raw data supporting the conclusions of this article will be made available by the authors, without undue reservation.

## Footnotes

^1^https://ourworldindata.org/coronavirus
