# Supplementary material for: Clinical features of COVID-19 infection in patients with myasthenia gravis: a real-world retrospective study
Source: Front Public Health. 2024 Aug 27;12:1421211. doi: 10.3389/fpubh.2024.1421211 (PMC11384569; doi:10.3389/fpubh.2024.1421211)
Supplement: Supplementary file 1 [file Table_1.DOCX]

Supplementary Material

Supplementary Table 1 Clinical features of 14 patients with severe or critical COVID-19

| Patient | Antibody | MG  duration | Maximal MGFA | Pre–COVID-19 MGFA-PIS | Comorbidities | Thymus | Pre–COVID-19 MG treatment | Affected muscles | Hospitalization  Duration(d) | Post–COVID-19 treatment | Anticoagulant | ICU  Duration(d) | Invasive ventilation(h) | Outcomes |
| --- | --- | --- | --- | --- | --- | --- | --- | --- | --- | --- | --- | --- | --- | --- |
| NO.1,71, M | AChR-Ab (+) | 1 | Ⅴ | Ⅱa | HBP, DM | Normal | N | Respiratory muscles | 43 | IVIG, IVMP, TZP | Y | Y (25) | Y (115) | Survival |
| NO.2,40, M | AChR-Ab (+) | 8 | Ⅴ | Ⅲa | No | Normal | N | ocular muscles  +Bulbar muscles | 27 | IVIG, IVMP, TZP | N | Y (27) | Y (353) | Survival |
| NO.3,76, M | AChR-Ab (+) | 10 | Ⅴ | Ⅱb | HBP, DM, CRC | Normal | N | Respiratory muscles | 20 | IVIG, IVMP, FOX | Y | N | N | Survival |
| NO.4,80, M | AChR-Ab (+) | 2 | Ⅴ | Ⅱb | HBP, CI | Normal | PED 7.5 mg; PB; TAC | Bulbar muscles+ Respiratory muscles | 1 | IVIG, IVMP, FOX | N | N | N | Death |
| NO.5,81, M | AChR-Ab (+) | 4 | Ⅲb | Ⅱa | DM | Normal | PB; TAC | Cervical muscles | 14 | IVIG, IVMP, FOX | N | N | N | Survival |
| NO.6,70, M | AChR-Ab (+) | 3 | Ⅲa | Ⅰ | HBP, CHD, AS, CI | Normal | PB | Cervical muscle+ Respiratory muscles | 11 | IVIG, IVMP, FOX | Y | N | N | Survival |
| NO.7,76, M | AChR-Ab (+) | 5 | Ⅳb | Ⅱa | CHD, CI | Normal | PED 20 mg; PB | Respiratory muscles | 13 | IVIG, IVMP, FOX | N | N | N | Survival |
| NO.8,77, F | AChR-Ab (+) | 1 | Ⅲb | Ⅰ | HBP, DM, CHD | Normal | N | Limb muscles | 11 | IVMP, TAC, FOX | Y | N | N | Survival |
| NO.9,78, F | Negative | 5 | Ⅲa | Ⅱ | DM, CHD, TB | Normal | PED 3.75 mg | Limb muscles | 9 | IVMP, CXM | Y | N | N | Survival |
| NO.10,76, F | Negative | 11 | Ⅳb | Ⅱa | HBP, DM | Normal | PB | Bulbar muscles+ Respiratory muscles | 13 | IVIG, IVMP, FOX | Y | N | N | Survival |
| NO.11,54, F | AChR-Ab (+) | 2 | Ⅴ | Ⅱa | No | Normal | PED 20 mg; PB | Respiratory muscles | 20 | IVIG, IVMP, TZP | Y | Y (14) | Y (259) | Survival |
| NO.12,46, F | AChR-Ab (+) | 11 | Ⅳb | Ⅱb | No | Removal | PED 5 mg; PB | Respiratory muscles | 11 | IVIG, IVMP, TZP | N | Y (11) | Y (3) | Death |
| Patient | Antibody | MG  duration | Maximal MGFA | Pre–COVID-19 MGFA-PIS | Comorbidities | Thymus | Pre–COVID-19 MG treatment | Affected muscles | Hospitalization  Duration(d) | Post–COVID-19 treatment | Anticoagulant | ICU  Duration(d) | Invasive ventilation(h) | Outcomes |
| NO.13,74, F | AChR-Ab (+) | 3 | Ⅴ | Ⅱb | HBP, DM | Normal | PED 5 mg; PB | Respiratory muscles | 4 | IVIG, IVMP, TZP | Y | Y (4) | Y (22) | Death |
| NO.14,61, M | AChR-Ab (+) | 7 | Ⅲb | Ⅲb | DM | Normal | BCDT | Limb muscles+ Respiratory muscles | 14 | IVMP, BCDT, TZP, FNC | N | N | N | Survival |

Abbreviations: MG, myasthenia gravis;COVID-19,Coronavirus disease 2019;MGFA,Myasthenia Gravis Foundation of America; PIS, post-intervention Status；PED, prednisone; HBP, high blood pressure; DM, diabetes mellitus; CRC, colorectal cancer; CI, cerebral infarction; AS, asthma; CHD, coronary heart disease; TB, tuberculosis; TAC, tacrolimus; PB, pyridostigmine bromide; BCDT, B cell depleting therapy; IVIG, intravenous immunoglobulin; IVMP, intravenous methylprednisolone; TZP, piperacillin–tazobactam; FOX, cefoxitin; CXM, cefuroxime; FNC, azvudine

(Continued)
